# A comparison of alternative strategies for choosing control populations in observational studies

**DOI:** 10.1007/s10742-014-0135-8

**Published:** 2015-01-30

**Authors:** Adam Steventon, Richard Grieve, Jasjeet S. Sekhon

**Affiliations:** 1 Department of Health Services Research and Policy, London School of Hygiene and Tropical Medicine, Keppel Street, London, WC1E 7HT UK; 2 The Health Foundation, 90 Long Acre, London, WC2E 9RA UK; 3 Travers Department of Political Science and Department of Statistics, UC Berkeley, 210 Barrows Hall #1950, Berkeley, CA 94720-1950 USA

**Keywords:** Program evaluation, Quasi-experiments, Propensity score matching

## Abstract

Various approaches have been used to select control groups in observational studies: (1) from within the intervention area; (2) from a convenience sample, or randomly chosen areas; (3) from areas matched on area-level characteristics; and (4) nationally. The consequences of the decision are rarely assessed but, as we show, it can have complex impacts on confounding at both the area and individual levels. We began by reanalyzing data collected for an evaluation of a rapid response service on rates of unplanned hospital admission. Balance on observed individual-level variables was better with external than local controls, after matching. Further, when important prognostic variables were omitted from the matching algorithm, imbalances on those variables were also minimized using external controls. Treatment effects varied markedly depending on the choice of control area, but in the case study the variation was minimal after adjusting for the characteristics of areas. We used simulations to assess relative bias and means-squared error, as this could not be done in the case study. A particular feature of the simulations was unexplained variation in the outcome between areas. We found that the likely impact of unexplained variation for hospital admissions dwarfed the benefits of better balance on individual-level variables, leading us to prefer local controls in this instance. In other scenarios, in which there was less unexplained variation in the outcome between areas, bias and mean-squared error were optimized using external controls. We identify some general considerations relevant to the choice of control population in observational studies.

**Electronic supplementary material:**

The online version of this article (doi:10.1007/s10742-014-0135-8) contains supplementary material, which is available to authorized users.

## Introduction

Well-conducted randomized controlled trials (RCTs) are often considered the gold standard in comparative effectiveness research as they can balance both observed and unobserved variables between treatment groups. However, for many examples in health services and outcomes research, RCTs are infeasible and the best available information on effectiveness comes from an observational study. These must be designed and analyzed carefully so that findings are not biased by differences in the characteristics of patients or settings (Rubin 2010). While techniques such as instrumental variable estimation can handle confounding due to unobserved as well as observed characteristics (Stukel et al. 2007), valid instruments are rare, so instead studies tend to use approaches that assume no unobserved confounders. For example, propensity scores can be used to select, from a wider population of potential controls, a matched subgroup that is similar to the intervention group with respect to observed variables (Rosenbaum and Rubin 1983). Matching methods are appealing because, for some estimands, regression models are more robust to model specification when applied to matched rather than unmatched data (Ho et al. 2007).

Many advances have been made in analytical methods for observational studies. For example, genetic matching uses computer-intensive search algorithms to find more closely balanced matched control groups than traditional approaches using the propensity score (Sekhon and Grieve 2012). Also, doubly robust methods can provide unbiased estimates when either the treatment selection or the outcome model is correctly specified (Bang and Robins 2005). On the other hand, relatively little attention has been paid to study design (Rubin 2007). This is an important omission as improvements in design could reduce the main threat to the validity of observational studies, namely confounding due to unobserved variables. One design issue that has received scant attention relates to the choice of higher-level unit from which the control group is selected. The issue arises because interventions are often piloted within a sample of units, such as hospitals, geographic areas or schools. Within these units, a subset of individuals will receive the intervention and others the control (e.g., usual practice). However, often data are available for other potential control units. Therefore, investigators have a choice at the design stage of a study between selecting matched controls from within the intervention areas (or, more generally, higher-level units), from other areas, or nationally. If selecting controls from other areas, these units could be selected as part of a convenience sample, or matched to the characteristics of the intervention areas. Studies have used the full range of approaches (McConnell et al. 2008; Nelson 2012; Roland et al. 2012).

In theory, the choice of control area can have complex implications for confounding at both the individual and area levels. While selecting controls from within an intervention area will automatically give perfect balance on area-level variables, it will not always give good balance on individual-level variables, as there may be limited overlap between the characteristics of treated and untreated individuals in the intervention area (Stuart and Rubin 2008). In situations of limited overlap, selecting controls externally may give better balance on both observed and unobserved individual-level variables than selecting controls from within the intervention area. To illustrate this hypothesis, suppose that, within the intervention area, a particular characteristic (older age) is associated with treatment receipt. In this situation, the local untreated group will contain relatively few older people, as these have been disproportionately recruited into the intervention. By contrast, there may be a relatively high number of older people in areas not offering the intervention, making it easier to obtain good matches on that variable when using external rather than local controls. The same argument applies to unobserved variables (such as extent of social support), but because unobserved variables cannot be taken into account by commonly-used analytical approaches, they are particularly important to balance by design. Based on these considerations, it is not clear which strategy for selecting control areas is optimal, but we might expect the optimal strategy to depend on the extent of confounding at the individual level versus area level. This reasoning was used by Griswold and Localio (2010) in an observational analysis of the effect of concurrent use of proton-pump inhibitors and clopidogrel, and led them compare an approach using local controls with approaches using national controls and controls from similar hospitals. They found that local controls produced worse balance on observed individual-level variables, but they could not assess unobserved variables, bias, or statistical efficiency.

While careful selection of control areas has long been recognized as crucial for case–control studies (Miettinen 1985), little methodological research has been undertaken to guide the choice of control population in cohort studies, which are the focus of this paper. Meta-epidemiological work has found that observational studies tend to give treatment effects that are more similar to those from RCTs when their control group is sourced locally rather than from a matched area (Glazerman et al. 2003). Discrepancies in treatment effects tend to be larger still when the observational study uses a convenience sample of areas or takes controls from a national sample. Meta-epidemiological work can control for only a limited number of study characteristics (Deeks et al. 2003), but some studies have controlled for research setting more closely by comparing randomized and observational studies that share a single treatment group. A review of these also concluded that local controls should be preferred (Cook et al. 2008), though the number of constituent studies was small and dominated by labor market interventions (Shadish et al. 2008). These reviews did not examine whether the relative benefit of local versus external control groups varies between alternative research settings with different levels of confounding at the local and area levels. Furthermore, although RCTs are often considered to be the gold standard in comparative effectiveness research, in some cases there are legitimate reasons why observational studies should give different treatment effects to RCTs, relating to measurement, study samples, or interventions (Hartman et al. forthcoming).

Several research methods use multiple control groups to assess bias, but these do not address the prior question about which control area should be preferred. For example, Campbell (1969) proposed using multiple control groups to put bounds on treatment effects, and also to confirm that the variation in treatment effects is as expected given prior information about the different groups (this approach is known as ‘control by systematic variation’). Rosenbaum (1987) gave a detailed account of how multiple control groups could be used to test for unobserved confounding at the individual level, though he did not consider area-level confounding. Lu and Rosenbaum (2004) considered the situation of one treatment group and two control groups, and developed a matching algorithm to make three pairwise comparisons while optimizing the use of individuals. Multiple control groups are sometimes used in the medical literature, for example by comparing treated patients to both historic and concurrent controls (Harrison et al. 2010), but they seem to be rare in comparison to studies that use a single group (Austin 2008).

As few studies have assessed the implications of the choice of control population on bias and mean-squared error (MSE), we conducted a simulation study. We calibrated these simulations to a case study of an intervention that aimed to reduce unplanned hospital admissions for older people, and then varied the assumptions made for the propensity score model and the response model across a total of 45 scenarios. Although we expected local controls to be preferable in many scenarios, the simulation reports that in some settings a strategy of selecting external controls gives both lower bias and lower MSE. This paper provides general methodological recommendations to inform the choice of control populations in future studies. We also append R code to provide practical tools for investigators to undertake simulations at an early stage of study design to determine which control population is likely to give the least bias in their research setting. These simulations could complement the use of multiple control groups, if these are available, by identifying which control group is likely to give the most reliable inferences.

This paper is organized as follows. First, we describe the estimands typically of interest in a cohort study and the various ways in which the choice of control area can affect bias. Then, in Sect. 3, we describe the case study and show how balance and estimated treatment effects depend on the choice of control area. Section 4 describes the simulation design and sets out a number of scenarios with varying levels of individual-level and area-level confounding. The results of the simulation study are given in Sect. 5, and the final section concludes.

## Statistical considerations relating to the choice of control area

Following the Rubin causal model (1978), we begin by positing two potential outcomes for each individual, $Y(1)$ and $Y(0)$, relating to outcomes under intervention and control, respectively. A common target estimand is the Sample Average Treatment effect for the Treated (SATT), defined as the average difference between these potential outcomes over the group of people receiving the intervention (Imai et al. 2008). Common approaches to estimate the SATT assume no unobserved confounding. In other words, if $Z$ indicates assignment to the treatment, then we assume that there is a set of observed baseline variables $X$ such that:

1 $$Y(1), Y(0)\coprod {Z | X}$$

If this assumption is valid, then an unbiased estimate of the treatment effect at each level of $X$ can be obtained by calculating the differences in the observed responses of intervention and control patients at that level (Rosenbaum and Rubin 1983). Therefore, an unbiased estimate of SATT can be produced by selecting a matched control group with the same distribution of $X$ as the intervention group. This can be accomplished by matching on a single scalar quantity known as the propensity score, which reflects the probability of treatment assignment, conditional on observed variables (Rosenbaum and Rubin 1983). A complementary approach, genetic matching, uses a computer-intensive search algorithm to find the matches that maximize balance across all variables in $X$, given the data (Sekhon and Grieve 2012).

Bias can arise when matching for several reasons. First, the matching algorithm may not produce groups with the same distribution of observed variables. Second, some important baseline variables may be unobserved, and so omitted from the matching algorithm. We decompose the estimation error into these two components by supposing an additive model for the outcome of each individual (Imai et al. 2008):

$$Y_{i} (t) = g_{t} (X_{i} ) + h_{t} (U_{i} )$$

Here, $g_{t}$ and $h_{t}$ are, in general, unknown functions ($t = 0,1$), and $U$ represents the unobserved baseline variables. Following 1–1 matching, the estimation error is:

$$\begin{aligned} SATT - D & = \frac{1}{n}\sum\limits_{{i \in \{ i|Z_{i} = 1\} }} {\left\{ {g_{1} (X_{i} ) + h_{1} (U_{i} ) - g_{0} (X_{i} ) - h_{o} (U_{i} )} \right\}} \\ & \quad - \left\{ {\frac{1}{n}\sum\limits_{{i \in \{ i|Z_{i} = 1\} }} {\left\{ {g_{1} (X_{i} ) + h_{1} (U_{i} ) } \right\}} - \frac{1}{n}\sum\limits_{{i \in \{ i|Z_{i} = 0\} }} {\left\{ {g_{0} (X_{i} ) + h_{0} (U_{i} )} \right\}} } \right\} \\ \end{aligned}$$

or:

$$\frac{1}{n}\sum\limits_{{i \in \{ i|Z_{i} = 0\} }} {\left\{ {g_{0} (X_{i} ) + h_{0} (U_{i} ) } \right\}} - \frac{1}{n}\sum\limits_{{i \in \{ i|Z_{i} = 1\} }} {\left\{ { g_{0} (X_{i} ) + h_{o} (U_{i} )} \right\}}$$

Here, $D$ is the estimator, and $n$ is the number of individuals in the intervention group, which is the same as the number of individuals in the matched control group because the matching is 1–1.

The set of observed baseline variables $X$ contains some variables at the individual level ($X_{i, 1}$) and some at the area level ($X_{i, 2}$), and likewise for the unobserved baseline variables, $U$. We assume that the outcome model can be further decomposed into additive subcomponents relating to observed and unobserved variables at the individual and area levels, and thus write, for example:

$$g_{t} (X_{i} ) = g_{t}^{1} (X_{i,1} ) + g_{t}^{2} (X_{i,2} ).$$

where $g_{t}^{1}$ and $g_{t}^{2}$ are, in general, unknown functions that represent the subcomponents of the outcome model relating to observed variables at the individual and area levels, respectively ($t = 0,1$), and likewise for the unobserved variables at these levels, $h_{t}^{1}$ and $h_{t}^{2}$.

With this decomposition, there are four terms to the estimation error, representing the effects of imbalance on observed individual-level variables, unobserved individual-level variables, observed area-level variables, and unobserved area-level variables, respectively:

$$\frac{1}{n}\sum\limits_{{i \in \{ i|Z_{i} = 0\} }} {g_{0}^{1} (X_{i,1} )} - \frac{1}{n}\sum\limits_{{i \in \{ i|Z_{i} = 1\} }} {\left\{ {g_{0}^{1} (X_{i,1} )} \right\}}$$

$$\frac{1}{n}\sum\limits_{{i \in \{ i|Z_{i} = 0\} }} {h_{0}^{1} (U_{i,1} )} - \frac{1}{n}\sum\limits_{{i \in \{ i|Z_{i} = 1\} }} {\left\{ { h_{0}^{1} (U_{i,1} )} \right\}}$$

$$\frac{1}{n}\sum\limits_{{i \in \{ i|Z_{i} = 0\} }} {g_{0}^{2} (X_{i,2} )} - \frac{1}{n}\sum\limits_{{i \in \{ i|Z_{i} = 1\} }} {\left\{ { g_{0}^{2} (X_{i,2} )} \right\}}$$

$$\frac{1}{n}\sum\limits_{{i \in \{ i|Z_{i} = 0\} }} {h_{0}^{2} (U_{i,2} )} - \frac{1}{n}\sum\limits_{{i \in \{ i|Z_{i} = 1\} }} {\left\{ { h_{0}^{2} (U_{i,2} )} \right\}}$$

We now consider the situation in which there is one intervention area (containing both treated and untreated individuals) and several external areas (containing only untreated patients). Many of the issues would be the same if there were multiple intervention areas, a point we return to in Sect. 6.

If controls are selected from within the intervention area (forthwith referred to as ‘local controls’), then the third and fourth error terms will be zero, as both observed and unobserved area-level variables will be balanced automatically by design. However, the first error term will be nonzero when the matching algorithm is not able to balance the observed variables, $X_{i, 1}$. This can happen when there is poor overlap between the characteristics of treated and untreated individuals, as might be expected when the $X_{i, 1}$ are strong confounders (i.e., when they are strongly predictive of treatment assignment and outcome) or when a high proportion of local individuals receive the treatment (i.e., high intervention saturation). The second error term will in general be nonzero because matching algorithms cannot balance unobserved variables, except to the extent to which they are correlated with the observed variables that the matching algorithm is required to balance.

We explore three other strategies that may produce better balance on the individual baseline variables than local controls. These three other strategies use controls external to the intervention area, so the third and fourth error terms may be nonzero. However, individuals with the characteristics likely to lead to treatment assignment in the intervention area will not have been lost from the supply of potential controls in external areas. Thus, all other things being equal, overlap will be higher when controls are selected externally rather than locally. In turn, this can result in smaller errors through terms one and two.

Strategy 2 represents a commonly used, but perhaps ill-advised, approach whereby control areas are selected as part of a convenience sample, with little attention placed on the characteristics of the control areas. In the case study that follows, we implement this strategy a large number of times to show the substantial variability that arises in terms of balance and treatment effects. Strategy 3 is a better approach whereby the control area is matched to the intervention area with respect to the observed area-level variables $X_{i,2}$, thus minimizing the third error term, but not necessarily the fourth error term. The final strategy (strategy 4) is a national approach in which controls are taken from all areas external to the intervention area (i.e., a national sample). This approach maximizes the number of potential controls, which may result in closer matches on individual-level variables at the expense of worse balance on area-level variables than strategy 1.

The subsequent case study and simulation contrast these strategies across situations that typically arise in health services and outcomes research.

## Case study: rapid response service for older people

A rapid response service was introduced into a large, rural, district county area of England, as part of a national program to improve partnership working across care sectors (the Partnership for Older People Projects, or POPPs) (Windle et al. 2009). An important objective of the rapid response service was to prevent unplanned hospital admissions for older people through prompt treatment close to the individual’s home. However, a previous evaluation using controls from matched geographic areas found that the service had the opposite effect and increased these admissions, perhaps because of additional health needs identified by the rapid response team (Steventon et al. 2011, 2012).

We focus on the subset of older people (aged 70 or over) who were enrolled into the rapid response service during October 2008 with a history of hospital admissions (n = 108). We examine the effect of the service on the likelihood of participants having one or more unplanned hospital admission during the 12 months following enrollment. As in the original study, we obtained individual-level variables from the Hospital Episode Statistics (HES), which is a national database containing details of all hospital care funded by the National Health Service in England. Unlike the original study, we obtained these variables for people in each of the 33 district counties in England. Thus, we were able to apply the various strategies for control area selection described in Sect. 2.

Individual-level variables included: age; sex; socioeconomic deprivation score (defined at a small-area level^1^); diagnoses of four specific health conditions; total number of chronic health conditions; numbers of prior planned and unplanned hospital admissions; and a predictive risk score. The predictive risk score was an estimate of the probability of one or more unplanned hospital admission during the 12 months following enrollment, under usual care. It was based on an existing predictive risk model, with coefficients reweighted to match the patterns of hospital utilization that we observed for untreated individuals in the intervention area (Billings et al. 2006). We applied these reweighted coefficients to people in the other district counties to calculate their risk scores. Of all the variables, the predictive risk score, age and number of prior unplanned hospital admissions were the most strongly predictive of the outcome.

Strategy 2 was repeated 32 times (once for each of the district counties in England, excluding the intervention area). The matched geographic area for strategy 3 was selected according to an established method that is used to produce comparative statistics (Office for National Statistics 2010). This involved minimizing the Euclidean distance from the intervention area with respect to a standard set of 43 area-level variables, relating to: population age structure; population density; ethnic mix; average household size and structure; education; overall rates of long-term illness; transport; overall employment rates; and the prevalence of various occupations. The method was similar to that used to select the matched geographic area in the original study (Steventon et al. 2011), except that we used a wider set of variables. We implemented strategy 4 by pooling potential controls from all 32 counties.

For each strategy, we applied the study inclusion criteria to define a pool of potential controls who were aged 70 or over in October 2008 and had a history of hospital admissions. To remove the possibility that differing population sizes influenced results, we reduced the eligible population of each area to a random sample of 500. As 108 individuals received the intervention, this left 392 potential controls for strategy 1, producing a saturation of just less than 30 %. The number of potential controls for strategies 2 and 3 was 500, while in strategy 4 it was 16,000.

Within each of the chosen areas, we selected matched controls at the individual level using genetic matching (Sekhon and Grieve 2012). Matched controls were selected on a 1–1 basis with replacement, as this will typically lead to better balance on individual-level variables than matching without replacement, or 1–n matching (n > 1). Balance before and after matching was assessed using the standardized difference, defined as the difference in sample means as a proportion of the pooled standard deviation (Austin 2009). Although covariate balance should ideally be maximized without limit, a standardized difference of more than ±10 % has been used to denote meaningful imbalance (Normand et al. 2001). We report estimated treatment effects using the absolute risk difference (i.e., difference in proportions) and the relative risk difference, together with 95 % confidence intervals produced using methods that recognized the dependencies within matched data (Agresti and Min 2004).^2^

### Case study results

The three most prognostic variables (predictive risk score, age and number of prior unplanned admissions) had lowest standardized differences before matching when the control population was defined using a matched geographic area (strategy 3). For example, age had a standardized difference of 49.2 % before matching in strategy 3, compared with 56.9 % in strategy 1 (Table 1). By contrast, the socioeconomic deprivation score had lowest standardized difference when controls came from within the intervention area.

**Table 1:** Standardized differences before and after matching (%), with all individual baseline variables included in the genetic matching

	Strategy 1: Local controls (392 potential controls)		Strategy 2: Random areas (500 potential controls)^a^		Strategy 3: Matched area (500 potential controls)		Strategy 4: National (16,000 potential controls)	
	Before	*After*	Before	*After*	Before	*After*	Before	*After*
Mean predictive risk score	59.6	*4.7*	55.9	*4.5*	55.5	*4.7*	56.6	*1.3*
Mean age	56.9	*4.9*	51.7	*5.4*	49.2	*4.8*	53.3	*2.7*
Mean number of unplanned admissions^b^	48.5	*3.3*	42.5	*1.6*	37.5	*0.0*	43.2	*0.0*
Female gender	22.5	*−6.0*	24.8	*0.0*	30.4	*−2.0*	26.1	*0.0*
Mean socioeconomic deprivation score	−1.5	*1.3*	21.8	*7.5*	13.8	*6.6*	20.1	*0.7*
Cancer prevalence	−3.2	*2.6*	5.2	*2.6*	2.6	*7.8*	2.5	*0.0*
Diabetes prevalence	3.5	*5.7*	11.3	*0.0*	6.2	*2.8*	9.4	*0.0*
Congestive heart failure prevalence	13.0	*0.0*	11.0	*3.0*	8.5	*3.0*	11.3	*0.0*
Ischemic heart disease prevalence	11.5	*−12.1*	12.8	*0.0*	16.5	*4.8*	13.4	*0.0*
Mean number of chronic conditions	26.0	*−4.6*	25.3	*0.6*	24.1	*3.5*	24.1	*1.2*
Mean number of planned admissions	13.2	*9.0*	14.6	*6.5*	11.9	*3.9*	14.6	*0.0*
Mean (absolute) standardized difference	23.6	*4.9*	25.9	*5.4*	23.3	*4.0*	25.0	*0.5*

Negative values imply that the variable was lower on average in the intervention than matched control group

^a^For reasons of space, standardized differences for strategy 2 are the medians over all 32 possible geographies. However, there was substantial variation depending on the choice of geography. Age, for example, showed standardized differences that ranged from 38.3 to 66.6 % before matching, depending on which area was chosen, and from −12.5 to 20.0 % after matching. Ranges for the predictive risk score were from 46.7 to 61.3 % before matching, and from 0.4 to 12.5 % after matching. For the number of unplanned admissions, ranges were from 34.7 to 48.3 % before matching, and from −9.9 to 11.5 % after matching

^b^Admission counts are over the year prior to enrollment

**Fig. 1:**
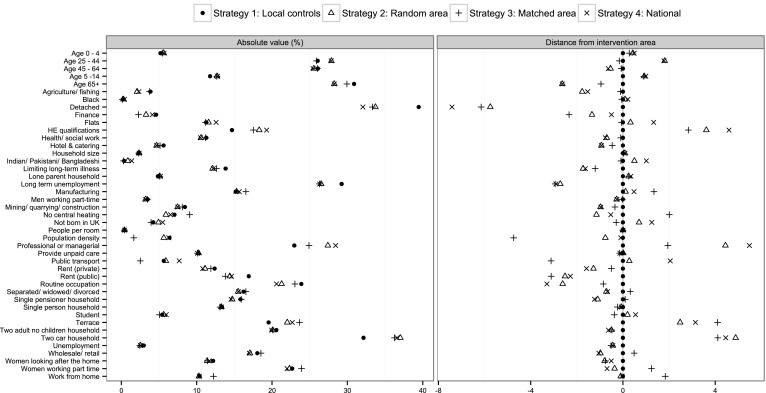
Area-level variables. The *first panel* shows the values of each of the 43 area-level variables under each strategy, while the *second panel* shows relative differences from the intervention area. Figures for Strategy 2 (‘random area’) are medians over all 32 possible geographies. Figures for Strategy 4 (‘national’) are weighted means over the 32 geographies (weighted for population size)

After matching, strategy 4 (national controls) gave the best balance across all observed individual-level variables, reflecting the larger population size. For example, age had a standardized difference of 2.7 % under national controls (Table 1), compared with 4.9 % when using local controls. However, the intervention area was different from the national sample in terms of area-level variables, such as the proportion of residents aged 65 or over (Fig. 1). Although strategy 3 (matched area) reported higher standardized differences than strategy 4 at the individual level (e.g., 4.8 vs. 2.7 % for age), it nonetheless outperformed local controls (average standardized difference 4.0 vs. 4.9 %). Furthermore, the matched area was more like the intervention area than the national sample (Fig. 1).

As expected, estimated treatment effects from strategy 2 (convenience sample) were very sensitive to the area chosen, and relative risk ratios ranged from 1.35 (95 % CI 1.00–1.82) to 3.87 (2.43–6.16). However, estimated treatment effects for strategies 1, 3, and 4 were very similar, with relative risk ratios of 2.07, 1.93 and 2.07, respectively (Table 2).

**Table 2:** Estimated treatment effects

	Strategy 1: Local controls	Strategy 2: Random areas	Strategy 3: Matched area	Strategy 4: National
Relative risk (95 % confidence interval)				
With all baseline variables included in the genetic matching	2.07 (1.46–2.94)	Median: 2.23 (1.52–3.28) Minimum: 1.35 (1.00–1.82) Maximum: 3.87 (2.43–6.16)	1.93 (1.37–2.73)	2.07 (1.47–2.92)
With age and predictive risk score omitted from the genetic matching	2.76 (1.89–4.03)	Median: 2.42 (1.61–3.63) Minimum: 1.66 (1.21–2.27) Maximum: 3.87 (2.32–6.44)	2.32 (1.57–3.42)	2.23 (1.55–3.21)
Absolute risk difference (95 % confidence interval)				
With all baseline variables included in the genetic matching	0.28 (0.27–0.29)	Median: 0.30 (0.28–0.31) Minimum: 0.14 (0.13–0.15) Maximum: 0.40 (0.39–0.41)	0.26 (0.25–0.27)	0.28 (0.27–0.29)
With age and predictive risk score omitted from the genetic matching	0.34 (0.33–0.35)	Median: 0.31 (0.30–0.33) Minimum: 0.21 (0.20–0.23) Maximum: 0.40 (0.39–0.41)	0.31 (0.29–0.32)	0.30 (0.28–0.31)

### Inducing unobserved confounding

As we hypothesized that the relative strengths of the strategies will depend on the extent of unobserved confounding at the individual level, we repeated the analysis after omitting two important prognostic variables from the genetic matching algorithm, namely age and predictive risk score.^3^ Thus, we treated these variables as being unobserved. After matching, standardized differences on these variables were high across all strategies, but they were lower when using a matched control area than when using local controls, reflecting the generally better balance that existed before matching. Standardized differences for age were 44.3 and 49.4 %, after matching, in these two strategies, respectively.^4^ Standardized differences for the predictive risk score were 27.4 and 31.3 %, respectively.

As would be expected, each strategy reported higher estimated treatment effects when the two prognostic variables were omitted from the matching algorithm. However, these increased by less when controls were sourced from a matched control area than with local controls (Table 2).

The case study findings suggest that, all other factors being equal, using external control groups can lead to lower standardized differences on observed variables than local controls. Furthermore, estimated treatment effects were more robust to the unobserved confounding considered when matched controls came from a matched area rather than locally. Although external controls lead to better balance on individual-level variables, they also introduce the possibility of confounding due to area-level differences. We could not quantify the impact of this phenomenon in the case study, though the variability of the treatment effects observed under strategy 2 suggests it may be substantial. We now use simulations, calibrated to these data, to assess the implications of control area strategy for relative bias and statistical efficiency across a range of scenarios.

## Design of the simulation study

The simulations generalized previous examples (Drake 1993) to allow for observed and unobserved confounding at both individual and area levels (Table 3). One individual-level variable ($$x_{1,1}$$) was observed, while another ($$x_{2,1}$$) was unobserved. Two area-level variables ($$x_{1,2}$$ and $$x_{2,2}$$) were observed, while a third one ($$x_{3,2} )$$ was unobserved. We considered this to be the minimum number of covariates needed to test a sufficiently wide range of scenarios.

**Table 3:** The simulation design and relationships assumed in the base case scenario

	Level	Observation	Structure	Strength of relationship with intervention assignment ($\alpha )$	Strength of relationship with outcome ($\beta )$
$x_{1,1}$	Individual	Observed	$x_{1,1}$ and $x_{2,1}$ are correlated	0.5	0.30
$x_{2,1}$	Individual	Unobserved		Range 0.1–0.3	0.15
$x_{1,2}$	Area	Observed	Independent	n/a	0.01
$x_{2,2}$	Area	Observed	Determines the mean of $x_{2,1}$	n/a	0.05
$x_{3,2}$	Area	Unobserved	Independent	n/a	0.06

In designing the simulations, we were mindful that researchers often have access to data at the aggregate level, but not to their individual-level counterparts. In the case study, for example, we had access to socioeconomic deprivation scores defined at a small area level, but not to the person-level equivalents. Similarly, in the sensitivity analysis that omitted age from the genetic matching algorithm, a control area was still matched to the intervention area on the overall age distribution. To mimic this situation, we assumed that the mean of the unobserved individual-level variable ($x_{2,1}$) differed between areas, but that this mean value corresponded to the value of the observed area-level variable $x_{2,2}$. Thus, control areas could be selected to minimize differences in the distribution of $x_{2,1}$ between the intervention and control area.

### Generating baseline data

The three area-level variables ($x_{1,2}$–$x_{3,2}$) were generated for each of 49 geographic areas by sampling from independent standard normal distributions (i.e., mean 0, variance 1, pairwise correlations 0). Additionally, we assumed values of $x_{1,2}$ = $x_{2,2}$ = $x_{3,2}$ = 1 for the intervention area. This meant that, under the response models that are described below, the intervention area had atypical outcomes under control, as seems reasonable for areas that intervene to affect these outcomes. The assumption also meant that the intervention area had an atypical distribution of the unobserved individual-level covariate, $x_{2,1}$ (i.e., mean 1 as opposed to the expected level of 0), as would in general be the case.

The individual-level variables $x_{1,1}$ and $x_{2,1}$ were then generated for 1,000 individuals in each of the 50 areas, by sampling from a bivariate normal distribution, with means of 1 and $x_{2,2}$, respectively, a common variance of 1, and correlation 0.2.

Following previous simulation studies (Drake 1993; Austin et al. 2007), we assumed that the probability of receiving the intervention (the true propensity score) was a logistic function of the individual-level variables:

$$\Pr (t = 1 |x_{1,1} ,x_{2,1} ) = [ 1 + \exp \{ - (\alpha_{0} + \alpha_{1,1} x_{1,1} + \alpha_{2,1} x_{2,1} )\} ]^{ - 1}$$

Here, $\alpha_{1,1}$ and $\alpha_{2,1}$ controlled how predictive the two individual-level variables were of intervention assignment, and therefore one aspect of confounding. The final coefficient ($\alpha_{0}$) could be calibrated so that, in expectation across repeated simulations, a given proportion ($N\%$) of the individuals in the intervention area would receive the intervention.

### Forming matched control groups

After baseline data had been generated, the different strategies to select control populations were applied. The matched control area for strategy 3 was selected as the one that minimized the Euclidean distance from the intervention area with respect to the two observed area-level variables ($x_{1,2}$ and $x_{2,2}$). Although the Mahalanobis metric could have been used (Rosenbaum and Rubin 1985), the result would have been the same as $x_{1,2}$ and $x_{2,2}$ were independent by assumption. The unobserved area-level variable $x_{3,2}$ could not be taken into account when selecting the matched control area. The individual-level variables were also not taken into account, on the assumption that, in health services and outcomes research, individual baseline data are typically not collected until after the study areas have been chosen. The control area for strategy 2 (convenience sample) was selected at random, while strategy 4 pooled potential controls from across the 49 non-intervention areas.

Matched control groups were formed at the individual level under each of the strategies. This was done before outcome data were simulated, so that matching was blind to outcome (Rubin 2008). The relative simplicity of the simulation design (in particular, with regard to the number of covariates) meant that, unlike in the case study, genetic matching was not required to balance the observed characteristics. Instead, the propensity score was estimated by applying logistic regression to data from the intervention area, and matches were formed using nearest neighbor matching on this estimated propensity score (1–1, with replacement). Since $x_{2,1}$ was unobserved, it was omitted from the propensity score model, and the model contained only a single variable, $x_{1,1}$ (more generally, this variable might represent a weighted vector of many variables). When using external controls, the coefficients from the local propensity score model were applied to individuals in the control area.^5^

Balance on the individual-level baseline variables was assessed under each strategy by reporting standardized differences. We also report mean values of the area-level variables.

### Generating outcomes and assessing treatment effects

A dichotomous outcome was simulated according to a response model used in previous simulation studies (Drake 1993):

$$\begin{gathered} Pr(Y = 1 |x_{1,1} ,x_{2,1} ,x_{1,2} ,x_{2,2} ,x_{3,2} ,t) \hfill \\ \quad \quad \quad = [1 + \exp \{ - (\beta_{0} + \beta_{1,1} x_{1,1} + \beta_{2,1} x_{2,1} + \beta_{1,2} x_{1,2} + \beta_{2,2} x_{2,2} + \beta_{3,2} x_{3,2} + \delta t)\} ]^{ - 1} \hfill \\ \end{gathered}$$

The coefficients labeled $\beta_{i,j}$ determined how predictive the baseline variables were of the outcome. The binary variable, $t$, indicated whether the individual received the intervention ($t$ = 1) or not ($t$ = 0). The true intervention effect was denoted by $\delta$ and assumed to be zero.

Intervention effects were estimated under each strategy using the absolute risk difference (difference in proportions), relative to the corresponding matched control group. Bias was calculated by comparing the estimated treatment effect with the true intervention effect (*i.e.*, zero). We report mean bias over 20,000 simulations. We also obtained the MSE by squaring the difference between the estimated and true intervention effects and averaging over all simulations.

### Calibration of the simulation study

The simulations were calibrated to the HES data by taking unplanned hospital admission to be the outcome, as in the case study, and then conducting sensitivity analysis for the response model and for the true propensity score model. In calibrating the level of individual confounding under the base case scenario, we assumed that the unobserved confounder was less predictive of intervention assignment and outcome than the observed confounder, and adopted $\alpha_{1,1}$ = 0.5, $\alpha_{2,1}$ = 0.2, $\beta_{1,1}$ = 0.3 and $\beta_{2,1}$ = 0.15. See Online Resource 1 for a derivation of these values using national HES data.

An important aspect of the simulation design concerned the amount of explained and unexplained variation in outcomes between areas (this variation being generated through $x_{1,2} , x_{2,2}$ and $x_{3,2}$). To calibrate this aspect of the simulations, we assessed to what extent the risk of unplanned hospital admission varies between similar individuals living in different areas of England, again using national HES data. Such between-area variation was assessed using the median odds ratio (MOR) (Larsen and Merlo 2005), which is defined as the odds ratio that would be expected, in median, between people with the same individual-level variables selected from two randomly-chosen areas. The MOR was calculated as 1.08 for people aged 70 or over in England—see Online Resource 1.

We made a conservative assumption about the amount of area-level variation that was explained by the observed area-level variables. Thus, we calibrated the simulation to two specific area-level variables, namely overall socioeconomic deprivation score and overall hospital admission rate (leading to $\beta_{1,2}$ = 0.01 and $\beta_{2,2}$ = 0.05). The remainder of the variation was assumed to be unexplained. A preliminary simulation showed that setting $\beta_{3,2}$ to approximately 0.06 gave an MOR of 1.08, and that approximately 70 % of the resulting variation was unexplained (i.e., due to the unobserved variable).^6^

We calibrated the intercept of the true propensity model ($\alpha_{0}$) to give an intervention saturation ($N\%$) of 30 %, as in the case study.

### Scenarios tested

We compared the bias and MSE resulting from each of the strategies under the following scenarios for the response model and for the true propensity score model.

Scenarios for the response model were:

1. As described above, ‘base case’ scenario was calibrated to the associations seen for unplanned hospital admissions in HES data. Thus, $\beta_{1,1}$ = 0.3, $\beta_{2,1}$ = 0.15, $\beta_{1,2}$ = 0.01, $\beta_{2,2}$ = 0.05, $\beta_{3,2}$ = 0.06, and MOR = 1.08.
2. The ‘simple confounding’ scenario assumed no confounding except through the observed individual-level variable ($\beta_{2,1}$ = $\beta_{1,2}$ = $\beta_{2,2}$ = $\beta_{3,2}$ = 0, $\beta_{1,1}$ = 0.3). This was the ideal situation, under which all of the evaluation designs were expected to perform well.
3. The ‘no area-level variation’ scenario assumed no systematic variation in outcomes between areas, other than through the individual-level variables ($\beta_{1,2}$ =$\beta_{2,2}$ = $\beta_{3,2}$ = 0). Thus, the MOR was 1.
4. The ‘no unexplained area-level variation’ scenario assumed that all variation in outcomes between areas could be explained ($\beta_{1,2}$ = 0.01, $\beta_{2,2}$ = 0.05,$\beta_{3,2}$ = 0). Individual-level confounding was the same as in the base case.
5. The ‘high unexplained area-level variation’ scenario targeted a higher MOR of 1.3, which required $\beta_{3,2}$ = 0.3. Other coefficients were the same as in the base case, implying that 95 % of the area-level variation was unexplained.

Scenarios for the true propensity score model were:

- The central assumption, that $\alpha_{1,1}$ = 0.5, $\alpha_{2,1}$ = 0.2 and $N\%$ = 30 %.
- Lower and higher saturation ($N\%$), of 10 % and 50 %.
- Lower and higher confounding through the unobserved individual-level variable, i.e. $\alpha_{2,1}$ equal to 0.1 and 0.3.

Finally, we repeated the simulations with a normally distributed, rather than dichotomous, outcome, and when matching without replacement, rather than with replacement.

## Results of the simulation study

### Standardized differences

The matching algorithm was generally able to find matched control groups that were closely balanced on the observed individual-level variable, regardless of which strategy was used to define the control population. For example, in strategy 1 (local controls), the matched control group had a mean of 1.352 on the observed variable, versus 1.354 for the intervention group in the base case scenario, leading to a standardized difference of 0.23 % (Table 4). Increasing the saturation increased the standardized difference under strategy 1, as the supply of potential control patients from within the local area became more limited. The standardized difference also increased under this strategy when the unobserved person-level variable became more predictive of intervention status. This led to greater before-matching differences on the observed variable, because of the correlation assumed between the individual-level variables. Although standardized differences were low under strategy 1, selecting controls from other areas could reduce them still further. Standardized differences were no more than 0.13 % under strategies 2 and 3, and less than 0.01 % under strategy 4.

**Table 4:** Balance in observed person-level confounder, $x_{1,1}$

*N*%	$\alpha_{2,1}$	Means (SDs) after matching					Standardized differences (%) after matching			
		Treated	Matched controls from strategy:				Strategy:			
			(1) Local controls	(2) Random areas	(3) Matched area	(4) National	(1) Local controls	(2) Random areas	(3) Matched area	(4) National
10	0.1	1.455 (0.099)	1.453 (0.099)	1.454 (0.099)	1.454 (0.099)	1.455 (0.099)	0.18	0.12	0.12	0.00
	0.2	1.471 (0.100)	1.469 (0.099)	1.470 (0.099)	1.470 (0.099)	1.471 (0.100)	0.19	0.13	0.13	0.00
	0.3	1.486 (0.098)	1.484 (0.098)	1.484 (0.098)	1.484 (0.098)	1.486 (0.098)	0.20	0.13	0.13	0.00
*30*	0.1	1.344 (0.056)	1.342 (0.056)	1.343 (0.056)	1.343 (0.056)	1.344 (0.056)	0.22	0.07	0.07	0.00
	*0.2*	*1.354* (*0.056*)	*1.352* (*0.056*)	*1.353* (*0.056*)	*1.354* (*0.056*)	*1.354* (*0.056*)	*0.23*	*0.08*	*0.07*	*0.00*
	0.3	1.363 (0.056)	1.361 (0.056)	1.362 (0.056)	1.362 (0.056)	1.363 (0.056)	0.24	0.08	0.07	0.00
50	0.1	1.244 (0.044)	1.241 (0.043)	1.243 (0.043)	1.243 (0.043)	1.244 (0.044)	0.30	0.05	0.05	0.00
	0.2	1.250 (0.043)	1.247 (0.043)	1.250 (0.043)	1.250 (0.043)	1.250 (0.043)	0.31	0.05	0.05	0.00
	0.3	1.256 (0.043)	1.253 (0.043)	1.255 (0.043)	1.255 (0.043)	1.256 (0.043)	0.33	0.05	0.05	0.00

Italic part shows the base case scenario

Each of the approaches for selecting the control population led to large imbalances on the unobserved individual-level variable ($x_{2,1}$), especially when this variable was strongly predictive of intervention status (Table 5). Strategy 1 (local controls) produced a standardized difference of 19.40 % under the base case scenario, whereas using strategy 3 (matched control area) resulted in a smaller standardized difference, of 18.14 %. The relative advantage of strategy 3 over strategy 1 increased with higher saturation and stronger confounding; at low saturation levels (10 %), strategy 1 produced the lower standardized differences. Using controls from random areas or from a national sample produced very large standardized differences on the unobserved variable across all scenarios.

**Table 5:** Balance in unobserved person-level confounder, $x_{2,1}$

*N*%	$\alpha_{2,1}$	Means (SDs) after matching					Standardized differences (%) after matching			
		Treated	Matched controls from strategy:				Strategy:			
			(1) Local controls	(2) Random areas	(3) Matched area	(4) National	(1) Local controls	(2) Random areas	(3) Matched area	(4) National
10	0.1	1.176 (0.101)	1.080 (0.110)	0.097 (1.004)	1.040 (0.251)	0.090 (0.201)	9.66	109.03	13.76	109.64
	0.2	1.261 (0.100)	1.071 (0.109)	0.094 (1.003)	1.046 (0.251)	0.094 (0.202)	19.30	118.23	21.81	118.31
	0.3	1.345 (0.100)	1.062 (0.109)	0.093 (1.010)	1.047 (0.251)	0.100 (0.201)	28.74	127.31	30.27	126.61
*30*	0.1	1.132 (0.058)	1.036 (0.076)	0.071 (0.998)	1.015 (0.240)	0.068 (0.164)	9.63	106.74	11.75	107.03
	*0.2*	*1.198* (*0.058*)	*1.006* (*0.076*)	*0.072* (*1.005*)	*1.018* (*0.238*)	*0.071* (*0.165*)	*19.40*	*113.90*	*18.14*	*113.99*
	0.3	1.260 (0.057)	0.976 (0.077)	0.071 (0.996)	1.022 (0.237)	0.072 (0.165)	28.91	121.01	24.26	120.93
50	0.1	1.094 (0.045)	0.998 (0.075)	0.040 (1.005)	1.001 (0.238)	0.048 (0.157)	9.65	106.03	9.29	105.21
	0.2	1.139 (0.044)	0.948 (0.075)	0.062 (1.003)	0.998 (0.235)	0.050 (0.158)	19.36	108.91	14.31	110.20
	0.3	1.183 (0.044)	0.900 (0.075)	0.052 (0.997)	0.998 (0.238)	0.051 (0.156)	28.83	115.30	18.93	115.41

Italic part shows the base case scenario

While strategy 1 (local controls) exactly balanced the three area-level variables ($x_{1,2} , x_{2,2}$ and $x_{3,2}$) strategy 3 could only balance the two observed area-level variables in expectation (mean 1.0 and standard deviation 0.2, compared with a value of 1.0 in the local area). Strategy 3 could not balance the unobserved area-level variable (mean 0, standard deviation 1). Strategies 2 and 4 led to large imbalances on all area-level variables.

### Bias and mean-squared error

**Fig. 2:**
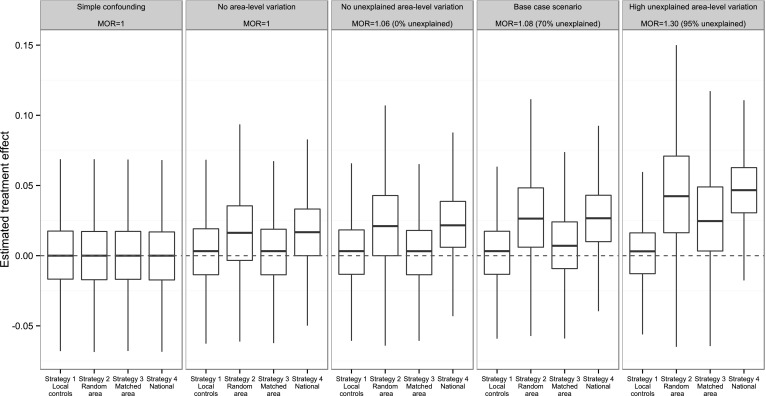
*Box plots* of the estimated treatment effects based on 20,000 replications from the simulation experiment. The *horizontal red line* represents the true treatment effect. Saturation = 30 % and $\alpha_{2,1} = 0.2$ throughout (Color figure online)

As would be expected, all strategies produced near-unbiased treatment effect estimates in the ‘simple confounding’ scenario, when the only confounder was the observed individual-level variable (Fig. 2, first panel; Table 6). When there was unobserved confounding at the individual level, but no area-level variation in outcomes (corresponding to an MOR of 1), using a matched control area still gave the least biased and most precise estimates (Fig. 2, second panel; Table 6). Similarly, when area-level variation in outcomes existed but was entirely explained by the observed variables, using a matched control area again produced the least biased estimates, though no longer the lowest MSE (Fig. 2, third panel). The final two scenarios shown in Fig. 2 include unexplained area-level variation in outcomes.

**Table 6:** Bias (mean-squared error) as a percentage of the population

	Strategy 1: Local controls	Strategy 2: Random areas	Strategy 3: Matched area	Strategy 4: National
Central assumptions (*N* = 30 %)				
Simple confounding	0.04 (6.51)	−0.02 (6.71)	0.02 (6.67)	−0.02 (6.50)
No area-level variation	0.26 (5.92)	1.64 (11.18)	0.24 (5.90)	1.68 (8.97)
No unexplained area-level variation	0.26 (5.61)	2.23 (14.97)	0.23 (5.71)	2.18 (10.89)
*Base case scenario*	*0.24* (*5.36*)	*2.73* (*17.69*)	*0.79* (*6.53*)	*2.66* (*13.14*)
High unexplained area-level variation	0.17 (4.49)	4.74 (41.39)	2.76 (19.69)	4.68 (27.79)
Sensitivity analysis: low saturation (*N* = 10 %)				
Simple confounding	−0.02 (19.49)	−0.05 (19.36)	0.01 (19.48)	0.03 (19.72)
No area-level variation	0.26 (16.86)	1.60 (22.76)	0.25 (17.26)	1.66 (21.10)
No unexplained area-level variation	0.25 (16.74)	2.19 (26.26)	0.29 (16.25)	2.18 (22.66)
Base case scenario	0.22 (15.53)	2.70 (29.17)	0.78 (17.28)	2.65 (24.64)
High unexplained area-level variation	0.20 (12.54)	4.59 (49.36)	2.69 (28.80)	4.56 (37.29)
Sensitivity analysis: high saturation (*N* = 50 %)				
Simple confounding	0.02 (4.06)	0.00 (4.07)	−0.01 (4.04)	−0.03 (4.06)
No area-level variation	0.26 (3.75)	1.66 (8.81)	0.20 (3.80)	1.65 (6.57)
No unexplained area-level variation	0.24 (3.51)	2.25 (13.06)	0.24 (3.61)	2.18 (8.59)
Base case scenario	0.23 (3.36)	2.74 (15.80)	0.74 (4.31)	2.70 (11.05)
High unexplained area-level variation	0.20 (2.73)	4.89 (41.92)	2.79 (18.49)	4.78 (26.57)

Italic part shows the base case scenario.$\alpha_{2,1}$ = 0.2 throughout

The base case scenario (Fig. 2, fourth panel) had an MOR of 1.08, with 70 % of this variation being unexplained. In this scenario, local controls gave the least biased and most precise estimates, with a bias of 0.24 %. Strategies 2 and 4 were very biased, whereas using a matched control area produced a bias closer to the local approach (0.79 %, see Table 6). The scenario with higher unexplained area-level variation (MOR = 1.30, 95 % unexplained), exaggerated the differences between the strategies still further (Fig. 2, last panel).

Strategies 2 and 4 gave large but similar biases in all except for the ‘simple confounding’ scenario, because neither strategy addressed the possibility that the unobserved individual-level variable might be distributed differently within the intervention area to without, and thus there were large imbalances on that variable under both strategies (Table 5). Estimates from strategy 4 were more precise than those from strategy 2 (Table 6).

Our conclusions for the base case scenario did not change when varying the amount of confounding through the unobserved individual-level variable ($\alpha_{2,1}$). Both strategies 1 and 3 reported higher levels of bias at higher values of this parameter ($\alpha_{2,1}$ = 0.3, bias 0.31 and 0.85 %, respectively), and lower biases at lower values ($\alpha_{2,1}$ = 0.1, bias 0.14 and 0.71 %, respectively). Matching without replacement marginally increased the standardized differences obtained for the observed individual-level variable when using local controls, but the impact on the overall bias was very small in the base case scenario (see Table A2 and Figure A1, Online Resource 2). Using a normally distributed outcome gave a similar pattern to Fig. 2 (see Figure A2, Online Resource 2).

## Discussion

Careful design is of paramount importance to observational studies since, however advanced the analytical method, the study is likely to be biased if the underlying assumptions are not met (Rubin 2008). Investigators have used a range of approaches to define the control population when evaluating healthcare interventions, but the relative benefits of some popular design choices (in particular, local or external control populations) have rarely been directly assessed (Rosenbaum 1987; Stuart and Rubin 2008). The findings of the case study and simulations can assist investigators in deciding on their strategy for control area selection.

In the case study, balance on individual-level variables was improved by using controls from a matched area rather than locally. When we induced unobserved confounding by omitting two prognostic variables from the matching algorithm (namely, age and predictive risk score), balance on these unobserved variables was also better when selecting controls from a matched area than when selecting controls locally. The simulations built upon the case study, and identified two criteria that were necessary for matched control areas to produce the best balance on individual-level variables. First, intervention saturation had to be relatively high—at least 30 %, the level seen in the case study. Second, the relationship between the unobserved variable and treatment assignment had to be relatively strong, as was the case for age and predictive risk score in the case study. The intuition behind the second condition is that, if the relationship between those variables was weak, then relatively good balance can be achieved locally. Meanwhile, selecting controls from outside of the intervention area risks systematic differences in the distribution of the unobserved individual-level variable and area-level variation in the outcome.

In the case study, treatment effects were more robust to induced, unobserved confounding when using a matched control area than local controls. The matched control area in the case study was selected using an established set of variables. We were reassured that, given the wide range of treatment effects that could be produced when using external areas (see Column 3, Table 2), treatment effects produced using the matched area were similar to those estimated locally. However, case study could not assess bias, which can arise from differences at the area level as well as at the individual level.

The simulations showed that a matched control area produced the lowest bias of all the strategies, provided that, first, it produces better balance at the individual level and, second, area-level variation either does not exist or can be largely explained by the observed area-level variables. In the terminology of Sect. 2, this means that error terms 1, 2 and 3 are minimized while error term 4 is close to zero. In other scenarios, where there was substantial unexplained variation in outcomes, a research design using a matched control area was more biased than one using local controls. In other words, the increases in error terms 3 and 4 associated with moving from local to external controls outweighed reductions in terms 1 and 2. For hospital admissions (represented by the base case scenario in Table 6), significant unexplained variation was likely, and so local controls gave least bias. Previous research has found that regional variation in hospital admission rates is partly due to differences in service design, admission thresholds and culture (Joynt and Jha 2012)—factors that are not often captured in routine data sources. Had we better data on area-level confounders, we would have been closer to the ‘no unexplained variation’ scenario than the ‘base case’ scenario, and thus might have preferred to use a matched control area.

Translating the results of the simulation back into the case study, we infer that the preferred estimate of the treatment effect relies on local not external controls. Thus, our preferred estimate of the relative risk of unplanned admissions is the local estimate, i.e. 2.07 (95 % CI 1.46–2.94). This information does not negate the value of using multiple control groups, as proposed by Campbell (1969) and Rosenbaum (1987). Indeed, under strategy 2, we repeated the matching algorithm 32 times, once for each potential choice of control population. Every analysis reported more unplanned hospital admissions among intervention than control patients, increasing the degree of confidence we place on this finding. However, precise effect sizes varied greatly depending on the choice of area, from a rate ratio of 1.35 to one of 3.87. The considerations described above lead us to prefer an estimate of around double. This estimate is likely to be least affected by unobserved confounding, but it is still susceptible to it.

### Limitations and future research

Although we considered a range of scenarios for both the response model and the propensity score model, the simulations were still limited in some respects. We assumed that the areas had the same population size whereas, if some areas were larger than others, then this would increase their attractiveness as sources of controls, all other factors being equal. We also assumed that the distribution of the unobserved individual-level variable $x_{1,2}$ differed between areas in ways that could be controlled for by careful selection of the control area. This reflected a common situation in which individual-level variables are manifested at area levels. For example, although individual education level might not be available, estimates might be available of the average education level of residents of different areas, perhaps from surveys. If such information is not available, then using a matched control area will generally not balance unobserved variables, as is apparent from the results of strategy 2 (Table 5). We also assumed that individual-level variables could not be used to select the matched control area. This reflects a common situation in which data can only be obtained from a small number of areas, either because of the cost of data collection or because of information governance considerations. However, in other situations, national individual-level data may be available from administrative data (Steventon et al. 2012) and could be used to select control areas.

We modeled the relatively simple situation in which there is a single intervention unit, and assumed that this was prone to atypical levels of the outcome under control, as would generally be the case. One could apply the same strategies 1–4 if there were several intervention units, but in general one would select several matched control areas under strategy 3. These areas could be selected by first constructing a propensity score that models the decision of each area to offer the intervention. Each intervention area would then be matched to a control area (for example, using nearest-neighbor matching on the propensity score or genetic matching), and then an individual-level matching algorithm would be applied to the corresponding area pairs. Other strategies also become available in this more general setting. Griswold and Localio (2010) fitted a single, multilevel propensity score model using individual-level data from several hospitals. This aimed to model both area-level and individual-level decision-making and resulted in a one-step matching process. These authors could not directly assess bias and statistical efficiency in their setting, but future work could conduct simulations similar to those in the current study to assess the statistical properties of their approach. Abadie et al. (2010) considered the same situation but created synthetic control groups by weighting the outcomes of several control areas. In their example, this approach gave good balance on the historical trend in the outcome. Stuart and Rubin (2007) considered a simpler situation with one intervention area and one external area. Although their method did not deal with unobserved confounding at the individual level, they made an adjustment for area-level group differences that could be developed for the more general scenario.

This paper addressed approaches to selecting control areas when estimating sample average treatment effects. If the estimand of interest was a population average treatment effect, then one would need to deal with many of the same issues about confounding at individual and area levels (Hartman et al. forthcoming). In estimating sample average treatment effects, we used a relatively simple matching method (nearest-neighbor matching) and a relatively simple estimator (difference in proportions). We also addressed scenarios in which observed individual-level variables were easily balanced, as is apparent from Table 4. If balance on these variables were harder to achieve, then more sophisticated matching methods (Hansen 2008; Sekhon and Grieve 2012) or more complex estimators (Bang and Robins 2005; Ho et al. 2007) may be helpful. However, these would not overcome unobserved confounding, and so we expect that our findings would hold true in the more general setting.

Variance estimation following matching was not the focus of the current paper but its importance for inference is recognized. The case study used an approach to estimating confidence intervals that reflected the paired nature of the matched data set (Agresti and Min 2004). Further work could incorporate recent developments in matching methods to allow for the dependencies in the data that arise as part of the matching process when matching with replacement, but also recognizing any clustering of individuals within control areas (Abadie and Imbens 2012).

Finally, we assumed that a standardized data set exists across areas, and so we did not take into account the possibility that measurement varied between areas. Such variation would introduce additional biases and uncertainty when selecting external controls. On the other hand, we did not account for spillover effects, whereby the intervention affects the care received by local untreated individuals. These might lead investigators to prefer external controls.

## Conclusions

Our findings underscore the importance of considering the data generating process underlying observational data sets, which is a multilevel phenomenon when patients come from more than one higher-level unit (e.g., from several geographic areas). The theory behind the use of propensity scores (Rosenbaum and Rubin 1983), when applied to a multilevel context, emphasizes the need to model correctly the process by which some areas offer the intervention and others do not, as well the process by which some individuals receive the intervention and others do not. This observation explains why large biases arose when using randomly chosen control areas or national controls. Investigators may trade off individual-level versus area-level confounding by selecting matched controls from a matched area rather than from within the intervention area. Box 1 summarizes some factors to consider when selecting control populations for observational studies.

**Box 1:** Factors to consider when selecting control populations

A situation in which local controls are preferred:
Low or moderate intervention saturation; and
Low risk of unobserved confounding at the individual level
A situation in which controls from a matched area are preferred:
High intervention saturation means there is a limited supply of controls from within the local area;
Unobserved confounding is likely at the individual level, and the unobserved confounder is a relatively strong predictor of treatment assignment;
The distribution of the unobserved confounder is likely to be similar in the matched control area to that in the intervention area; and
Area-level variation in outcomes either does not exist or can be largely explained by observed area-level variables that are accounted for in the matching
Other considerations include the relative population sizes of the areas, spillover effects and differences in measurement

Although there have been advances in analytical methods, design issues tend to be relatively neglected in observational studies, and there is limited guidance to help researchers improve study design and assess whether a data set is adequate to answer the questions being asked of it (Rubin 2010). We have provided a set of considerations that relate to the control population, and append code to help investigators undertake similar simulations to those presented here at the design stage of future observational studies, if there is doubt about which approach is best. This could complement sensitivity analyses using multiple control groups.

## Electronic supplementary material

Below is the link to the electronic supplementary material.

Supplementary material 1 (DOCX 1328 kb)

## Footnotes

1 Socioeconomic deprivation score was defined at the ‘Lower Super Output Area’ level, consisting of around 1,600 people on average, across all ages. In comparison, the county areas contained around 700,000 people on average. Therefore, socioeconomic deprivation score was treated as an individual-level variable, and attributed to individuals based on their place of residence.

2 Because we matched with replacement, an individual may have been selected as the control for several intervention patients. The 95 % CIs that we report do not allow for any dependency within the data from matching with replacement.

3 As age is one of the constituent variables of the predictive risk score, it was necessary to omit both variables.

4 Figures for predictive risk score are 44.6 % in strategy 3, compared with 58.0 % in strategy 1. Fuller data are available on request.

5 Although the propensity score model could have been fitted using potential controls from external areas, in this instance, the result would have been the same, as there was only a single variable in the empirical propensity score model.

6 To calculate the percentage of MOR that was due to observed variables, we calculated MOR when $\beta_{3,2}$ = 0, and expressed the resulting MOR as a proportion of total MOR.
