# Supplementary material for: A comparison of alternative strategies for choosing control populations in observational studies
Source: Health Serv Outcomes Res Methodol. 2015 Jan 30;15(3):157–81. doi: 10.1007/s10742-014-0135-8 (PMC4565881; doi:10.1007/s10742-014-0135-8)
Supplement: Supplementary file 1 — Supplementary material 1 (DOCX 1328 kb) [file 10742_2014_135_MOESM1_ESM.docx]

**Online resource 1: Calibration of the simulation study**

The simulations aimed to present a modest number of plausible scenarios to illustrate the strengths and limitations of the study designs, rather than be exhaustive in the range of scenarios covered. Therefore, although most applied studies allow for a large number of confounders, the simulation design was stylized and contained only two confounders at the individual level: $x_{1,1}$ (which was observed) and $x_{2,1}$ (which was unobserved). This appendix describes how the base case scenario was calibrated to national hospital data, in terms of these individual-level variables, the three area-level variables, and the intervention saturation. We took the outcome to be future unplanned hospital admission, as in the case study.

*Individual-level variables*

As one of the strongest predictors of future unplanned hospital admissions is the number of such admissions experienced in the past (Billings et al. 2006), for the purposes of calibration, $x_{1,1}$ was taken to be the number of admissions experienced in the 1-6 months before the intervention start date, while $x_{2,1}$ was taken to be the number of admissions experienced in the 7-12 months before. The scenario is, therefore, that the analysis is adjusted for the number of admissions occurring immediately before the intervention started, but not for the more distant history.

In the case study data, which concerned people aged 70 or over, the two person-level variables were observed to have correlation of 0.18. Both variables were associated with intervention assignment, with logistic regression coefficients of 0.25 and -0.12, respectively, after normalization. We assumed that the observed confounder was associated more strongly than this with treatment allocation (coefficient 0.5), as in practice several observed confounders would be adjusted for, and these would explain a greater proportion of the variation in intervention assignment than one variable would do itself. In the simulations, we set $\alpha_{1,1}$=0.5, and took various values for $\alpha_{2,2}$ (0.1, 0.2 and 0.3).

Both variables were associated with the outcome at the 5% significance level, with coefficients 0.29 and 0.14, respectively, after normalization. We took $\beta_{1,1}$= 0.3 and $\beta_{2,1}$=0.15.

*Area-level variation*

An important aspect of the simulation design concerned the amount of explained and unexplained variation in outcomes between areas (this variation being generated through the three area-level variables, $x_{1,2}, x_{2,2}$ and $x_{3,2}$). To calibrate this aspect of the simulations, we assessed to what extent the risk of unplanned hospital admission varies between similar individuals living in different areas of England, using national HES data. Rather than restrict our focus to the district councils considered in the case study (which tend to be predominately rural), we considered all of the 152 healthcare administrative areas that existed in 2008 ('Primary Care Trusts'). We restricted our attention to patients who were aged 70 or over with at least one recent admission, as in the case study. Between-area variation in unplanned hospital admissions in 2008 was assessed using the Median Odds Ratio (MOR) (Larsen and Merlo 2005), which is defined as the odds ratio that would be expected, in median, between people with the same individual-level variables selected from two randomly-chosen areas. The MOR was estimated using a random effects logistic model, adjusting for the individual-level variables that entered the predictive risk model used in the case study (Billings et al. 2006). The MOR was calculated as 1.08 for people aged 70 or over.

The area-level variables $x_{1,2}$ and $x_{2,2}$ were taken to be an area-level deprivation score and the overall hospital admission rate for the area, respectively. Both were associated with outcome (future unplanned hospital admission) at the 5% significance level, with coefficients 0.01 and 0.05, respectively, after normalization.

The role of the unobserved area-level confounder ($x_{3,2}$) was to capture unexplained variation in hospital admission rates between areas. A preliminary simulation revealed that setting $\beta_{3,2}$ = 0.06 would be expected to produce an MOR of 1.08 in the final simulated data set, given $\beta_{1,2}$=0.01 and $\beta_{2,2}$=0.05.

*Intervention saturation*

The final parameter used in the simulation study relates to the proportion of residents in the intervention area who received the intervention. In the case study, a total of 491 people aged 70+ with a history of hospital admissions eventually received the out-of-hours and daytime rapid response service, representing less than 1% of the total number of such people in the area. However, saturations of 20% and higher were modeled as it was assumed that researcher would have further narrowed down the population of potential controls (for example, by disease group). Higher saturations were tested as they are encountered in health services and outcomes research, and they helped to test the robustness of a method based on selecting controls from within the intervention area.

**Online resource 2: Sensitivity analysis**

This online resource contains sensitivity analysis when matching without replacement, and when using a normally distributed outcome.

**Matching without replacement**

The principal analysis presented in the main paper assumed that matching was conducted with replacement, so that a given individual could act as a matched control for more than one intervention patients. Matching without replacement marginally increased the standardized differences obtained for the observed individual-level variable when using local controls (see Table A1). It did not substantially impact standardized differences for the unobserved individual-level variable, or the overall bias (see Table A2 and Figure A1).

**Normally distributed outcome**

Figure A2 compares the box-and-whisker plots presented in the main paper with those produced using a normally distributed outcome. This had standard deviation 0.5 and mean equal to:

$$\left( \beta_{0}+\beta_{1,1}x_{1,1}+\beta_{2,1}x_{2,1}+\beta_{1,2}x_{1,2}+\beta_{2,2}x_{2,2}+\beta_{3,2}x_{3,2}+ \delta t \right)$$

The essential features of the original plot remains. As before, the “base case” scenario is calibrated to the case study ($\beta_{1,2}$=0.01, $\beta_{2,2}$=0.05, $\beta_{3,2}$=0.06, MOR=1.08). The second scenario assumes there is no confounding except through the observed individual variable ($\beta_{2,1}$=$\beta_{1,2}$=$\beta_{2,2}$=$\beta_{3,2}$=0) while, in the third, there is no area-level confounding ($\beta_{1,2}$=$\beta_{2,2}$=$\beta_{3,2}$=0). The final scenario assumes higher unexplained variation in outcomes between areas ($\beta_{3,2}$=0.3, MOR=1.3). Saturation = 30% and $\boldsymbol{\alpha}_{\boldsymbol{2,1}}\boldsymbol{=}$0.3 throughout.

**Table A1: Balance when matching with and without replacement (base case scenario with 30% saturation and** $\boldsymbol{\alpha}_{\boldsymbol{2,1}}\boldsymbol{=}$**0.3)**

|  |  | **Observed individual-level variable** | | **Unobserved individual-level variable** | |
| --- | --- | --- | --- | --- | --- |
|  |  | **With replacement** | **Without replacement** | **With replacement** | **Without replacement** |
| **Means (standard deviations)** | Treated | 1.363  (0.056) | 1.363 (0.055) | 1.260  (0.057) | 1.259 (0.057) |
|  | (1) Local controls | 1.361  (0.056) | 1.335 (0.049) | 0.976  (0.077) | 0.972 (0.057) |
|  | (2) Random areas | 1.362  (0.056) | 1.361 (0.055) | 0.071  (0.996) | 0.063 (1.012) |
|  | (3) Matched area | 1.362  (0.056) | 1.361 (0.055) | 1.022  (0.237) | 1.021 (0.235) |
|  | (4) National | 1.363  (0.056) | 1.363 (0.055) | 0.072  (0.165) | 0.073 (0.163) |
| **Standardized differences (%)** | (1) Local controls | 0.24 | 2.87 | 28.91 | 29.22 |
|  | (2) Random areas | 0.08 | 0.20 | 121.01 | 121.86 |
|  | (3) Matched area | 0.07 | 0.20 | 24.26 | 24.22 |
|  | (4) National | 0.00 | 0.00 | 120.93 | 120.79 |

**Table A2: Bias (mean-squared error) when matching with vs. without replacement (high unobserved area-level confounding scenario with 30% saturation and** $\boldsymbol{\alpha}_{\boldsymbol{2,1}}\boldsymbol{=}$0.3**)**

|  | **Strategy 1:**  **Local controls** | **Strategy 2: Random areas** | **Strategy 3:**  **Matched area** | **Strategy 4: National** |
| --- | --- | --- | --- | --- |
| **With replacement** | 0.30  (4.40) | 4.80  (42.29) | 2.83  (20.27) | 4.73  (28.28) |
| **Without replacement** | 0.33  (4.41) | 4.84  (42.67) | 2.82  (19.86) | 4.72  (28.09) |

Figure A1: Box plots of the estimated treatment effects based on 20,000 replications. The horizontal red line represents the true treatment effect. 30% saturation and $\alpha_{2,1}=$0.3.

Figure A2: Box plots of the estimated treatment effects based on 20,000 replications. The horizontal red line represents the true treatment effect. 30% saturation and $\alpha_{2,1}=$0.3.

**Online resource 3: R code for simulations**

library('MASS')

library('Matching')

library('lme4')

############################################

##### constants

############################################

# number of simulations

NSIM = 20000

# number of control areas (i.e., excluding intervention area)

NAREA = 49

# number of people in each area

NPOP = 1000

# number of iterations to perform to produce target saturation (set to a high number)

NSAT = 1000000

############################################

###### run code for the various sensitivity analyses considered in the paper

############################################

# compile all the functions below before running these!

main(N_run = 0.1, a2_run = 0.3)

main(N_run = 0.3, a2_run = 0.3)

main(N_run = 0.5, a2_run = 0.3)

main(N_run = 0.1, a2_run = 0.1)

main(N_run = 0.3, a2_run = 0.1)

main(N_run = 0.5, a2_run = 0.1)

main(N_run = 0.1, a2_run = 0.2)

main(N_run = 0.3, a2_run = 0.2)

main(N_run = 0.5, a2_run = 0.2)

###########################################

#### functions follow below

###########################################

# this is the main function that outputs arrays of balance statistics and estimated treatment effects

main <- function(N_run, a2_run) {

############################################

##### set assumptions

############################################

# set mean of area-level variables

amean <- c(0,0,0)

# set covariance matrix of x1,1 and x2,1

icov <- diag(2)

icov[1,2] <- 0.2

icov[2,1] <- 0.2

# set propensity score coefficients

a <- c(-1.7, 0.5, a2_run)

# set target saturation

N <- N_run

############################################

##### calibrate propensity score

############################################

# function returns saturation for given covariate

find_sat <- function(x) {

avar_m1 <- c(1,1,1)

ivar_m1 <- mvrnorm(n=NSAT, mu=c(1, avar_m1[2]), Sigma=icov)

p_m1 <- x + a[2]*ivar_m1[,1] + a[3]*ivar_m1[,2]

p_m1 <- exp(p_m1) / (1+exp(p_m1))

tr_m1 <- rbinom(NSAT, 1, p_m1)

mean(tr_m1)-N

}

# use R optimization routine to find the covariate value that produces target saturation in expectation

opt <- uniroot(find_sat, c(-10,10))

a[1] <- opt$root

############################################

##### conduct simulations

############################################

# this will contain estimated treatment effects

r_c <- NULL

# this will contain balance statistics (standardised differences)

b_c <- NULL

# loop through iterations of the data generating process

for(i in 1:NSIM) {

############################################

##### obtain matched data from all strategies

############################################

m <- getpairs(i, a, amean, icov)

# get number of matched intervention patients

n_matched <- sum(1*(m$tr==1)*(m$meth=="Method 1"))

# get balance statistics (standardised differences) under each strategy

b1 <- MatchBalance(tr~x1+x2, data = subset(m,m$meth=="Method 1"), ks = FALSE, nboots = 0, print.level = 0)

b2 <- MatchBalance(tr~x1+x2, data = subset(m,m$meth=="Method 2"), ks = FALSE, nboots = 0, print.level = 0)

b3 <- MatchBalance(tr~x1+x2, data = subset(m,m$meth=="Method 3"), ks = FALSE, nboots = 0, print.level = 0)

b4 <- MatchBalance(tr~x1+x2, data = subset(m,m$meth=="Method 4"), ks = FALSE, nboots = 0, print.level = 0)

# sort these out into a tidy array and add to b_c

compile <- function(df,meth) {

var <- c("x1", "x2")

sdiff <- c(df$BeforeMatching[[1]]$sdiff, df$BeforeMatching[[2]]$sdiff)

meanTr <- c(df$BeforeMatching[[1]]$mean.Tr,df$BeforeMatching[[2]]$mean.Tr)

meanCo <- c(df$BeforeMatching[[1]]$mean.Co, df$BeforeMatching[[2]]$mean.Co)

varTr <- c(df$BeforeMatching[[1]]$var.Tr, df$BeforeMatching[[2]]$var.Tr)

varCo <- c(df$BeforeMatching[[1]]$var.Co, df$BeforeMatching[[2]]$var.Co)

data.frame(var, sdiff, meanTr, meanCo, varTr, varCo, factor(meth), n_matched)

}

b <- rbind(compile(b1, "Method 1"), compile(b2, "Method 2"), compile(b3, "Method 3"), compile(b4, "Method 4"))

b$sim <- i

b_c <- rbind(b_c,b)

############################################

##### get estimated treatment effects under each scenario (for both binary and normally-distributed outcome)

############################################

r1 <- getresponse(

m=m,

b0 = 1.7,

b1 = 0.3,

b2 = 0.15,

b3 = 0.01,

b4 = 0.05,

b5 = 0.06,

delta = 0,

scen = "Base case",

i=i)

r2 <- getresponse(

m=m,

b0 = 1.7,

b1 = 0.3,

b2 = 0,

b3 = 0,

b4 = 0,

b5 = 0,

delta = 0,

scen = "Simple confounding",

i=i)

r3 <- getresponse(

m=m,

b0 = 1.7,

b1 = 0.3,

b2 = 0.15,

b3 = 0,

b4 = 0,

b5 = 0,

delta = 0,

scen = "No area-level variation",

i=i)

r4 <- getresponse(

m=m,

b0 = 1.7,

b1 = 0.3,

b2 = 0.15,

b3 = 0.01,

b4 = 0.05,

b5 = 0,

delta = 0,

scen = "No unexplained area-level variation",

i=i)

r5 <- getresponse(

m=m,

b0 = 1.7,

b1 = 0.3,

b2 = 0.15,

b3 = 0.01,

b4 = 0.05,

b5 = 0.3,

delta = 0,

scen = "High unexplained area-level variation",

i=i)

# combine estimated treatment effects from all scenarios

r <- rbind(r1, r2, r3, r4, r5)

r_c <- rbind(r_c, r)

}

############################################

# save output files

############################################

desc <- paste("N(",N,") ", "a2(", a[3], ")",

sep = "")

path <- "C:/" # update path for output files as appropriate

filename_r <- paste(path,"Response ", desc, ".csv", sep="")

filename_b <- paste(path,"Balance ", desc, ".csv", sep="")

filename_mor <- paste(path,"MOR ", desc, ".csv", sep="")

write.csv(r_c, filename_r)

write.csv(b_c, filename_b)

write.csv(estmor_c, filename_mor)

}

############################################

##### this function simulates outcome data and returns estimated treatment effects

############################################

getresponse <- function(m, b0, b1, b2, b3, b4, b5, delta, scen, i) {

############################################

##### first assume binary outcome

############################################

# simulate outcomes with actual treatment assignment

# note that, in the notation of the paper, b1-b5 are b1,1, b2,1, b1,2, b2,2, and b3,2, respectively

logit <- b0 + b1*m$x1 + b2*m$x2 + b3*m$x3 + b4*m$x4 + b5*m$x5 + delta*m$tr

logit <- exp(logit) / (1+exp(logit))

n <- nrow(m)

y <- rbinom(n, 1, logit)

# estimate treatment effects (using difference-in-means estimator)

eff_m1 <- mean(y[m$meth=="Method 1" & m$tr ==1]) - mean(y[m$meth=="Method 1" & m$tr ==0])

eff_m2 <- mean(y[m$meth=="Method 2" & m$tr ==1]) - mean(y[m$meth=="Method 2" & m$tr ==0])

eff_m3 <- mean(y[m$meth=="Method 3" & m$tr ==1]) - mean(y[m$meth=="Method 3" & m$tr ==0])

eff_m4 <- mean(y[m$meth=="Method 4" & m$tr ==1]) - mean(y[m$meth=="Method 4" & m$tr ==0])

meth <- c("Method 1", "Method 2", "Method 3", "Method 4")

eff <- c(eff_m1, eff_m2, eff_m3, eff_m4)

############################################

##### then with normally-distributed outcome

############################################

# simulate outcomes with actual treatment assignment

linear <- b0 + b1*m$x1 + b2*m$x2 + b3*m$x3 + b4*m$x4 + b5*m$x5 + delta*m$tr

n <- nrow(m)

y <- rnorm(n, linear, sd = 0.5)

# estimate treatment effects (using difference-in-means estimator)

eff_m1 <- mean(y[m$meth=="Method 1" & m$tr ==1]) - mean(y[m$meth=="Method 1" & m$tr ==0])

eff_m2 <- mean(y[m$meth=="Method 2" & m$tr ==1]) - mean(y[m$meth=="Method 2" & m$tr ==0])

eff_m3 <- mean(y[m$meth=="Method 3" & m$tr ==1]) - mean(y[m$meth=="Method 3" & m$tr ==0])

eff_m4 <- mean(y[m$meth=="Method 4" & m$tr ==1]) - mean(y[m$meth=="Method 4" & m$tr ==0])

meth <- c("Method 1", "Method 2", "Method 3", "Method 4")

eff_norm <- c(eff_m1, eff_m2, eff_m3, eff_m4)

############################################

##### finally return estimated treatment effects to main loop

############################################

data.frame(

meth = c(meth, meth),

type = c(rep("Binary",4), rep("Normal",4)),

eff = c(eff, eff_norm),

scen = scen,

sim = i)

}

############################################

##### this function produces matched pairs from all strategies

############################################

getpairs <- function(i, a, amean, icov) {

# generate area-level variables (x1,2-x3,2)

# (assumed independent)

avar <- mvrnorm(n=NAREA, mu=amean, Sigma=diag(3))

# select matched control area by minimizing Euclidean distance from the intervention area

# note values for intervention area are hardcoded as 1

t <- (avar-1)*(avar-1)

euclid <- t[,1] + t[,2]

match <- which(euclid == min(euclid))

# select random control area

area.random <- ceiling(runif(1, 0, NAREA-0.00001))

# compile area-level covariates for each strategy

# strategy 4 (national controls) is dealt with below

avar_m1 <- c(1,1,1) # strategy 1 (local controls)

avar_m2 <- avar[area.random,] # strategy 2 (random area)

avar_m3 <- avar[match,] # strategy 3 (matched area)

# generate individual-level covariates for all individuals in intervention area

ivar_m1 <- mvrnorm(n=NPOP, mu=c(1, avar_m1[2]), Sigma=icov)

# slightly more difficult for other three methods as we need to do all areas

ivar <- array(0, dim=c(NAREA,NPOP,2))

for(j in 1:NAREA) {

ivar[j,,] <- mvrnorm(n=NPOP, mu=c(1, avar[j,2]), Sigma=icov)

}

ivar_m2 <- ivar[area.random,,] # strategy 2 (random area)

ivar_m3 <- ivar[match,,] # strategy 3 (matched area)

ivar_m4 <- cbind(c(ivar[,,1]), c(ivar[,,2])) # strategy 4 (national)

# generate true propensity score

p_m1 <- a[1] + a[2]*ivar_m1[,1] + a[3]*ivar_m1[,2]

p_m1 <- exp(p_m1) / (1+exp(p_m1))

# these arrays will hold treatment assignments

ab <- NPOP*NAREA

tr_m1 <- rbinom(NPOP, 1, p_m1)

tr_m2 <- rep(0,NPOP)

tr_m3 <- rep(0,NPOP)

tr_m4 <- rep(0,ab)

# tidy up data by putting it into data frames (not strictly necessary)

# this is slightly more difficult for method 4 (national), as area-level covariates are not constant

data_m1 <- data.frame(tr = tr_m1, x1 = ivar_m1[,1], x2 = ivar_m1[,2], x3 = avar_m1[1], x4 = avar_m1[2], x5 = avar_m1[3])

data_m2 <- data.frame(tr = tr_m2, x1 = ivar_m2[,1], x2 = ivar_m2[,2], x3 = avar_m2[1], x4 = avar_m2[2], x5 = avar_m2[3])

data_m3 <- data.frame(tr = tr_m3, x1 = ivar_m3[,1], x2 = ivar_m3[,2], x3 = avar_m3[1], x4 = avar_m3[2], x5 = avar_m3[3])

data_m4 <- data.frame(

tr = tr_m4,

x1 = ivar_m4[,1],

x2 = ivar_m4[,2],

x3 = avar[ceiling((1:(NPOP*NAREA))/NPOP),1],

x4 = avar[ceiling((1:(NPOP*NAREA))/NPOP),2],

x5 = avar[ceiling((1:(NPOP*NAREA))/NPOP),3]

)

# fit empirical propensity score to data from intervention area, then apply coefficients to other areas

fm <- glm(tr~x1, data = data_m1, family=binomial(link="logit"))

data_m1$p_est <- fm$coeff[1] + fm$coeff[2] * data_m1$x1

data_m2$p_est <- fm$coeff[1] + fm$coeff[2] * data_m2$x1

data_m3$p_est <- fm$coeff[1] + fm$coeff[2] * data_m3$x1

data_m4$p_est <- fm$coeff[1] + fm$coeff[2] * data_m4$x1

data_m1$p_est <- exp(data_m1$p_est) / (1+exp(data_m1$p_est))

data_m2$p_est <- exp(data_m2$p_est) / (1+exp(data_m2$p_est))

data_m3$p_est <- exp(data_m3$p_est) / (1+exp(data_m3$p_est))

data_m4$p_est <- exp(data_m4$p_est) / (1+exp(data_m4$p_est))

############################################

# now we will form matched pairs

############################################

# select cases from intervention area

cases <- subset(data_m1,data_m1$tr==1)

# append cases to data frames for the three external strategies

data_m2_app <- rbind(data_m2,cases)

data_m3_app <- rbind(data_m3,cases)

data_m4_app <- rbind(data_m4,cases)

# do matching on 1-1 basis with replacement

m1 <- Match(Tr=data_m1$tr, X=data_m1$p_est, M=1, estimand = "ATT", replace = TRUE, ties = FALSE)

m2 <- Match(Tr=data_m2_app$tr, X=data_m2_app$p_est, M=1, estimand = "ATT", replace = TRUE, ties = FALSE)

m3 <- Match(Tr=data_m3_app$tr, X=data_m3_app$p_est, M=1, estimand = "ATT", replace = TRUE, ties = FALSE)

m4 <- Match(Tr=data_m4_app$tr, X=data_m4_app$p_est, M=1, estimand = "ATT", replace = TRUE, ties = FALSE)

# compile matched data sets under each strategy

match_m1 <- data_m1[c(m1$index.treated, m1$index.control),]

match_m2 <- data_m2_app[c(m2$index.treated, m2$index.control),]

match_m3 <- data_m3_app[c(m3$index.treated, m3$index.control),]

match_m4 <- data_m4_app[c(m4$index.treated, m4$index.control),]

# add pair ID

match_m1$pair <- rep(1:length(m1$index.treated),2)

match_m2$pair <- rep(1:length(m2$index.treated),2)

match_m3$pair <- rep(1:length(m3$index.treated),2)

match_m4$pair <- rep(1:length(m4$index.treated),2)

# produce combined data set of matched data from all strategies

match_m1$meth <- factor("Method 1")

match_m2$meth <- factor("Method 2")

match_m3$meth <- factor("Method 3")

match_m4$meth <- factor("Method 4")

match_all <- rbind(match_m1, match_m2, match_m3, match_m4)

match_all$sim <-i

# return combined data set to main loop

match_all

}
